# Locus of Control and Obesity

**DOI:** 10.3389/fendo.2014.00159

**Published:** 2014-10-07

**Authors:** Florence Neymotin, Louis R. Nemzer

**Affiliations:** ^1^Nova Southeastern University, Fort Lauderdale, FL, USA

**Keywords:** locus of control, obesity, leptin, HbA1c, MHLC

## Abstract

In the developed world, the hazards associated with obesity have largely outstripped the risk of starvation. Obesity remains a difficult public health issue to address, due in large part to the many disciplines involved. A full understanding requires knowledge in the fields of genetics, endocrinology, psychology, sociology, economics, and public policy – among others. In this short review, which serves as an introduction to the Frontiers in Endocrinology research topic, we address one cross-disciplinary relationship: the interaction between the hunger/satiation neural circuitry, an individual’s perceived locus of control, and the risk for obesity. Mammals have evolved a complex system for modulating energy intake. Overlaid on this, in humans, there exists a wide variation in “perceived locus of control” – that is, the extent to which an individual believes to be in charge of the events that affect them. Whether one has primarily an internal or external locus of control itself affects, and is affected by, external and physiological factors and has been correlated with the risk for obesity. Thus, the path from hunger and satiation to an individual’s actual behavior may often be moderated by psychological factors, included among which is locus of control.

Rates of obesity, along with its associated health-care costs, have exploded, leading to a critical topic of research regarding the pathways and risk factors involved. Of the currently identified determinants, co-occurring traits, and characteristics of individuals who have been diagnosed as obese, one that has not, to date, garnered quite as much attention as genetic or behavioral determinants is an individual’s perceived locus of control. Yet, an individual’s ability to make a decision and stick with it will be determined, in part, by their personality traits. While these personality traits could represent hormonal differences, they do not always need to.

In particular, the locus of control construct is defined as a person’s belief in the amount of control they have regarding specific events in their life. An internal locus refers to the belief that one’s behavior or stable personal characteristics controls specific events or outcomes, while an external locus of control refers to the belief that these events or outcomes are controlled by forces external to oneself (1).

Locus of control is an important characteristic in relation to obesity because, by definition, it indicates whether an individual believes that his or her environment and choices are under his or her control. Thus, in addition to actual physical cues of hunger or satiation, the ability to interpret those cues appropriately in a given social setting will help determine how obesity develops and persists. In the case of obesity in particular, it is more complicated to determine both the correct measure of locus of control to employ, as well as to definitively show a causal, rather than simply a correlational, relationship between locus of control and obesity (2, 3).

Locus of control has been variously defined as a sense of general, nutritional, or personal mastery (2, 4–6). Alternatively, a common measure is the multi-dimensional health locus of control (MHLC) (7). Autonomy and self-efficacy, similar in concept to locus of control, have also been identified as having a relationship with obesity (8–11). The plethora of definitions further complicates the search for the correct measure to use in establishing the appropriate and relevant relationship with obesity.

As noted, however, choosing the correct measure is only a part of the problem. While some authors have found no correlation, this was generally because of a paucity of data or issues with the measurement structure (11–13). The current consensus is that there should be at least a correlational relationship between some measure of locus of control – or another related concept – and obesity (14).

The next step is to determine whether locus of control simply happens, in most cases, to be more internal for thinner individuals, or whether the effect is causal in either direction. Proving the causality of this relationship does tend to be more complicated. Perhaps even more important is the direction of causation, with future interventions being much more effective if a more internal locus of control causes increased levels of obesity and not vice versa (5, 15–17). That said, it is also important to remember that locus of control is “mostly” considered to be a stable trait, and so it cannot easily be manipulated (18).

In order to address causality, one useful step would be to determine a theoretical structure for the mechanism by which internal locus of control may or may not help individuals reduce their risk for obesity. Literature taking this approach has followed one of several paths. Some authors have noted that an external locus generally relates more to depression and anxiety, making these individuals more likely to give up instead of persevering in the face of difficult tasks (19). Furthermore, these externally focused people, in principle, have higher levels of cortisol, very likely also affecting their long-term health (19). If cortisol simultaneously relates to stress and causes individuals to eat more and become obese, then, while the relationship between locus and control and obesity may be causal, the relationship between obesity and long-term health could represent a correlational one. This would be concerning indeed if cortisol were the only element at play, however, that is very likely not the case.

Another possibility for the way in which locus of control affects obesity is that externally focused individuals benefit more from the assistance provided by dietitians and health-care providers, as well as from the health-care providers themselves (20, 21). Specifically, internally focused people will tend to succeed more often in weight-loss endeavors than will the externally focused portions of the population (4, 8, 22–25). Also, there is some evidence to suggest that externally focused individuals receive poorer treatment from those same dietitians, and are afforded less patience and time focused on their health behaviors (20). As evidence for the opposite direction of causation, however, it is also possible that access to a good health-care provider will actually create a more internal locus of control, leading to better adherence to medication regimes, and/or exercise and nutritional schedules (21).

In addition to their interactions with health-care providers, relationships with peers, and parents in the case of children, can also be responsible for the relationship between locus of control and obesity (26, 27). Due to discrimination by others, a higher body mass index (BMI) should relate to a more internal locus of control (6). Interestingly, some work has found that women in these cases actually have a more internal sense of control, but the authors explain that this is likely a result of overcompensating behaviors. Generally, however, researchers do find a difference between men and women in peer groups affecting levels of obesity, and often explain these differences through personal control. Notice, however, that in these cases, the direction of causality is very likely reversed, with obesity causing a more external locus of control.

Research has also shown that peer groups can be positively employed in these more externally focused cases to create healthier habits and intentions (26). Similarly, parents can influence more externally focused children by discouraging sedentary behaviors or making them less available (28). Thus, creating interventions specific to individuals with varying loci of control is a very real possibility. Notice, however, that we are once again faced with the difficulty of disentangling the joint effects of exercise, obesity, and locus of control, since higher levels of activity may, in principle, simultaneously create a more internally focused individual, as well as a less obese one.

After establishing the posited links between locus of control and obesity from various other parts of the literature, we now turn to the medical and physiological aspects of the question at hand. In particular, clinical obesity is strongly influenced by a complex web of genetic, endocrine, and environmental components that provide the physiological and psychological basis for the sensations of hunger and satiation. These sensations are, in one sense, inchoate signals processed by the conscious faculties of the brain simultaneously under the influence of the higher level locus of control perceptions. Although complicated, a great deal of progress has been made in the last two decades in describing the hormonal circuitry that controls these feelings, along with the risk factors for unhealthy functioning of these processes (29). On a simple level, one may think of the “satiety” hormone leptin, produced by adipocytes (30) – and its “hunger” counterpart, ghrelin (31, 32), produced by the gastrointestinal tract (33), as being in competition for influencing the same set of receptors in the brain (34), especially the neuropeptide Y neurons in the hypothalamic arcuate nucleus (35). In 1994, the gene for leptin was first cloned (36), and falling leptin levels have been found to be a signal for starvation (37). On the other side, ghrelin, which increases feeling of hunger (38), is produced mostly by the stomach (39) as a response to a lack of food (40). Ghrelin levels are high prior to eating and lower afterward (41), thus transducing physical cues such as stomach distention into hormonal signals.

However, this simple picture obscures some important details. Since they have excess adipose tissue, obese individuals generally have higher levels of leptin (42) but, similar to the insulin resistance that is associated with type II diabetes, they do not exhibit a normal satiation response due to changes in the leptin detection circuit. Thus, leptin resistance is a strong risk factor for obesity (43), although the direction of causality is not totally uncontroversial (44). Leptin replacement therapy has, however, been found to be very effective in treating patients with dyslipidemia stemming from specific genetic mutations (45). This lends credibility to the point of view that obesity is not always the result of a lack of “willpower” or a specific worldview related to one’s personal locus of control. That is, in the cases of a congenital defect in the hunger/satiation neuronal circuitry due to a mutation, or the development of leptin resistance, negative health outcomes are difficult to avoid in the absence of specific interventions.

Type II diabetes, with its hallmark hyperglycemia, is an important comorbid condition with obesity. An indicator of average blood glucose levels is the percentage of glycated hemoglobin, denoted HbA1c. Glucose in the blood stream can become irreversibly bound to the hemoglobin of red blood cells. As a result, the fraction HbA1c of total hemoglobin is a proxy (46) for the average glucose (47) concentration over the previous 1–3 months. Obesity can also attenuate the effectiveness of drugs intended to reduce HbA1c (48). As a clinical outcome, HbA1c levels in patients with diabetes were predicted by their health loci of control (49). That is, people who credit factors other them themselves are likely to do less well (7).

In conclusion, the literature and experimental studies have documented the existence of a correlational relationship between locus of control and obesity. This relationship has not, in most of these studies, been tested for causality or directionality, so that proving more than the existence of a simple correlational relationship is still a much needed area for future research. This is a crucial area for future research because understanding exactly how obesity is affected in a physiological, environmental, and psychological fashion is an important first step to addressing the obesity epidemic that is increasing worldwide.

## Conflict of Interest Statement

The authors declare that the research was conducted in the absence of any commercial or financial relationships that could be construed as a potential conflict of interest.
